# Identification of the activity source of CO_2_ electroreduction by strategic catalytic site distribution in stable supramolecular structure system

**DOI:** 10.1093/nsr/nwaa195

**Published:** 2020-08-31

**Authors:** Sheng-Nan Sun, Ning Li, Jiang Liu, Wen-Xin Ji, Long-Zhang Dong, Yi-Rong Wang, Ya-Qian Lan

**Affiliations:** Jiangsu Collaborative Innovation Centre of Biomedical Functional Materials, Jiangsu Key Laboratory of New Power Batteries, School of Chemistry and Materials Science, Nanjing Normal University, Nanjing 210023, China; School of Chemistry and Chemical Engineering, Yangzhou University, Yangzhou 225002, China; Jiangsu Collaborative Innovation Centre of Biomedical Functional Materials, Jiangsu Key Laboratory of New Power Batteries, School of Chemistry and Materials Science, Nanjing Normal University, Nanjing 210023, China; State Key Laboratory of High-efficiency Coal Utilization and Green Chemical Engineering, Ningxia University, Yinchuan 750021, China; Jiangsu Collaborative Innovation Centre of Biomedical Functional Materials, Jiangsu Key Laboratory of New Power Batteries, School of Chemistry and Materials Science, Nanjing Normal University, Nanjing 210023, China; Jiangsu Collaborative Innovation Centre of Biomedical Functional Materials, Jiangsu Key Laboratory of New Power Batteries, School of Chemistry and Materials Science, Nanjing Normal University, Nanjing 210023, China; Jiangsu Collaborative Innovation Centre of Biomedical Functional Materials, Jiangsu Key Laboratory of New Power Batteries, School of Chemistry and Materials Science, Nanjing Normal University, Nanjing 210023, China

**Keywords:** strategic catalytic site distribution, supramolecular structure system, active source, CO_2_ electroreduction

## Abstract

Identification of the real catalytic site in CO_2_ reduction reaction (CO_2_RR) is critical for the rational design of catalysts and the understanding of reactive mechanisms. In this study, the catalytic activity of pyridine-containing materials was for the first time structurally demonstrated in CO_2_RR by crystalline supramolecular coordination compounds model system. The system consists of three stable supramolecular coordination compounds (Ni-TPYP, Ni-TPYP-1 and Ni-TPP) with different numbers (4, 2 and 0) of active pyridine groups (i.e. uncoordinated pyridine nitrogen atoms). The electrocatalytic test results show that with the decrease of the number of active pyridine groups, the CO_2_RR performance is gradually reduced, mainly showing the reduction of highest FE_CO_ (99.8%, 83.7% and 25.6%, respectively). The crystallographic, experimental and theoretical evidences prove that the CO_2_RR activity is more likely derived from uncoordinated pyridine nitrogen than the electrocatalytic inert metal nickel in porphyrin center. This work serves as an important case study for the identification of electrocatalytic activity of pyridine-containing materials in CO_2_RR by simple supramolecular model system.

## Teaser

The catalytic activity of pyridine-containing materials was for the first time structurally demonstrated in CO2 reduction reaction by supramolecular and coordination compounds structure model system including Ni-TPP, Ni-TPYP and Ni-TPYP-1.

## INTRODUCTION

The increasing anthropogenic CO_2_ emissions associated with fossil fuel consumption is the main culprit in global warming [1,2]. CO_2_RR achieved by renewable electricity is an elegant means to alleviate the above-mentioned issue, because it can convert CO_2_ into valuable products (such as CO, HCOOH, CH_4_, etc.) for efficient carbon cycling [3–11]. So far, the reported efficient electrocatalysts in CO_2_RR primarily focus on nanostructured materials [12,13] based on metal [14,15] (such as Cu [16–21], Co [22–24], Zn [25], Ni [26–30], etc.) or metal-free active sites (such as C [31], N [32–35], B [36,37], etc.). Among them, N-doped or N-heterocyclic nanostructured electrocatalysts have made great progress in product conversion and Faraday efficiency of CO_2_RR, but it is still very difficult to determine the active N sites in these electrocatalysts due to the lack of accurate and clear structural information [38]. Moreover, there are many other factors including defects [39–44], impurities and complicated components that can further affect the identification of the accurate active site [45]. In this case, crystalline electrocatalysts for CO_2_RR are believed to be good candidates for solving these issues, because their well-defined structures are most favorable for the identification of the catalytically active site and the study of the reactive mechanism [46,47]. Therefore, constructing a rational crystalline model system to verify the activity of N-containing electrocatalysts is very important to the development of CO_2_RR.

However, to date, there are very limited crystalline materials that are reported as electrocatalysts for CO_2_RR because of their relatively poor structural stability. Metal-porphyrin applied in CO_2_RR has many advantages [48]. Among them, the metal in the porphyrin center can serve as a catalytically active site for CO_2_RR and the rigid macrocyclic metalloporphyrin molecule with conjugated π-electron system is conducive to the migration of electrons [49,50]. More importantly, they have clear molecular structure and structurally adjustable properties, which are favorable for the study of reaction mechanism and reasonable optimization of catalytic performance [51,52]. Given these advantages, we hope to construct a simple and rational porphyrin-based model system that can be used for the identification of the electrocatalytic activity of N-containing nanomaterials in CO_2_RR.

Herein, we designed and synthesized three crystalline supramolecular coordination compounds containing different numbers of active pyridine groups (i.e. uncoordinated pyridine nitrogen atoms) to identify the electrocatalytic activity of pyridine-containing materials in CO_2_RR by controlling the catalytic sites. They are Ni-TPYP (TPYP = 5,10,15,20-tetra(3pyridiyl)-21H, 23H-porphine) with four uncoordinated pyridine N atoms, Ni-TPYP-1 with two uncoordinated pyridine N atoms as well as Ni-TPP (TPP = 5,10,15,20-tetraphenyl-21H,23H-porphine) with four inactive benzene groups, respectively. All of them can serve as crystalline heterogeneous electrocatalysts for CO_2_RR. At present, the metalloporphyrins with excellent CO_2_RR properties are mainly porphyrins containing Co, Fe and Cu [51,53,54], but no porphyrins with Ni [47] show excellent performance for CO_2_RR. Moreover, electrochemical inert Ni was chosen to reduce the electroactivity contribution of metal ion, because it has been demonstrated that high free energy is required for Ni to form the *COOH intermediate (an important intermediate during the process of CO_2_-to-CO conversion in CO_2_RR) in the rate-determining step (RDS) [47,55]. We thus studied the electrocatalytic CO_2_RR performance of Ni-TPYP, Ni-TPYP-1 and Ni-TPP. The experimental results show that as the number of uncoordinated pyridine N atoms decreases, the electrochemical activity and selectivity of these compounds exhibit such a tendency: Ni-TPYP > Ni-TPYP-1 > Ni-TPP. The Faraday efficiency for the CO_2_-to-CO conversion (FE_CO_) of Ni-TPYP, Ni-TPYP-1 and Ni-TPP is 99.8%, 83.7% and 25.6%, respectively. The crystallographic and experimental results clearly evidence that the superior electrocatalytic CO_2_RR performance of Ni-TPYP is mainly attributed to the uncoordinated active pyridine N atoms. Moreover, theoretical calculations also prove that CO_2_ reacts more readily with pyridine N than with metal Ni in porphyrin center.

## RESULTS AND DISCUSSION

**Figure 1.**
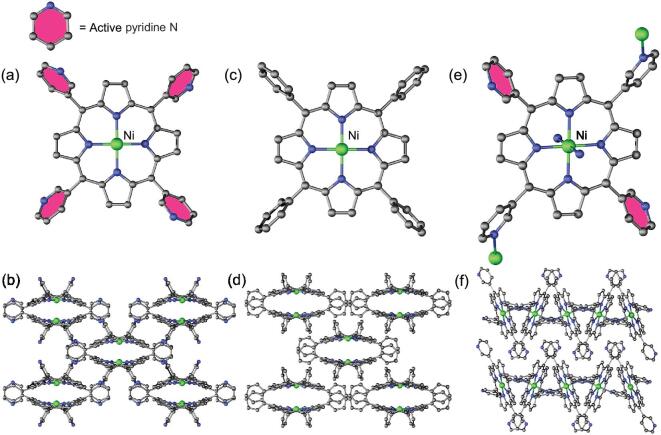
(a, c, e) The molecular structures of Ni-TPYP, Ni-TPP and Ni-TPYP-1. (b, d, f) The supramolecular stacked structures of Ni-TPYP, Ni-TPP and Ni-TPYP-1. Color code: C, black; N, blue; Ni, green.

Single-crystal X-ray diffraction analysis reveals that Ni-TPYP and Ni-TPP crystallize in the same tetragonal space group *I*-42*d* (Table S2 and Figs S1 and S2) and their structures as well as stacking patterns are almost identical. As shown in Fig. 1a and c, both compound molecule (Ni-TPYP and Ni-TPP) structures include a metal Ni ion and a pyridine/benzene-modified porphyrin ring (TPYP/TPP). The Ni^II^ ion connected with four N atoms from the porphyrin ring displays a square-planar coordination environment (Ni-TPP/Ni-TPYP). The pyridine N atoms of Ni-TPYP are naked. Both complexes further form similar 3D supramolecular structures by π-π stacking interaction and hydrogen bonds (Fig. 1b and d, and Fig. S3). Furthermore, no solvent molecules crystallize in their lattice. Ni-TPYP-1 crystallizes in monoclinic space group *P*2_1_*/c* (Table S2 and Fig. S4). Each Ni^II^ ion trapped in TPYP center coordinates two N atoms of pyridine from another two TPYP ligands to form a 2D layer and the layers are further stacked into supramolecular coordination compounds by π-π interaction (Fig. 1e and f, and Fig. S5). It is noted that each TPYP ligand in Ni-TPYP-1 still has two uncoordinated pyridine N atoms.

The phase purity of as-synthesized Ni-TPYP, Ni-TPYP-1 and Ni-TPP was determined by comparing their powder X-ray diffraction (PXRD) with simulated patterns derived from single crystal X-ray diffraction (Figs S7–S9). The pH stability was investigated by immersing the samples of Ni-TPYP, Ni-TPYP-1 and Ni-TPP into different pH aqueous solutions and 0.5 M KHCO_3_ aqueous solution ranging from 3 to 13. After 24 h, PXRD patterns before and after soaking show that no significant changes for each tested sample can be observed, confirming the structural integrity of Ni-TPYP, Ni-TPYP-1 and Ni-TPP under these conditions (Figs S10–S12). The thermal stability was also studied by thermogravimetric analysis (TGA) under O_2_ atmosphere and shown in Fig. S14.

**Figure 2.**
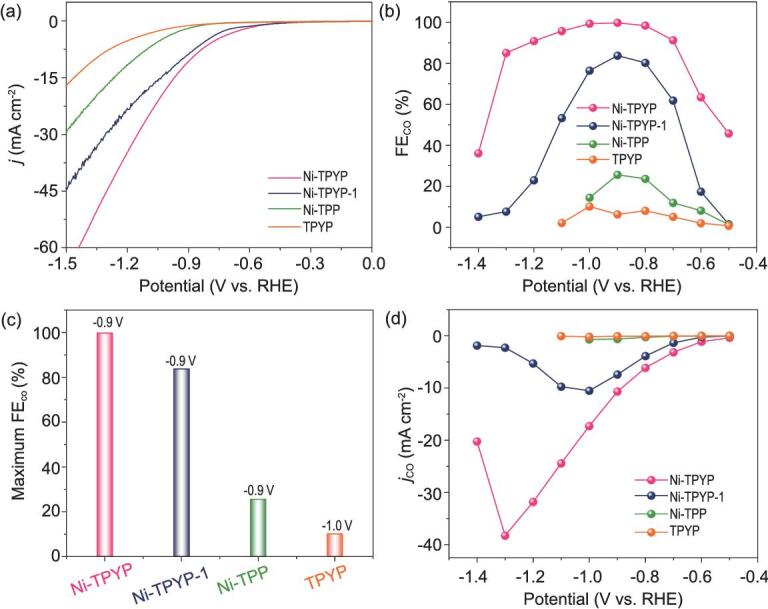
(a) LSVs of Ni-TPYP, Ni-TPYP-1, Ni-TPP and TPYP in CO_2_-saturated 0.5 M KHCO_3_ solution. (b) FE_CO_ of Ni-TPYP, Ni-TPYP-1, Ni-TPP and TPYP in CO_2_-saturated 0.5 M KHCO_3_ solution. (c) Maximum FE_CO_ of Ni-TPYP (−0.90 V), Ni-TPYP-1 (−0.90 V), Ni-TPP (−0.90 V) and TPYP (−1.00 V). (d) *j_CO_* of Ni-TPYP, Ni-TPYP-1, Ni-TPP and TPYP.

**Figure 3.**
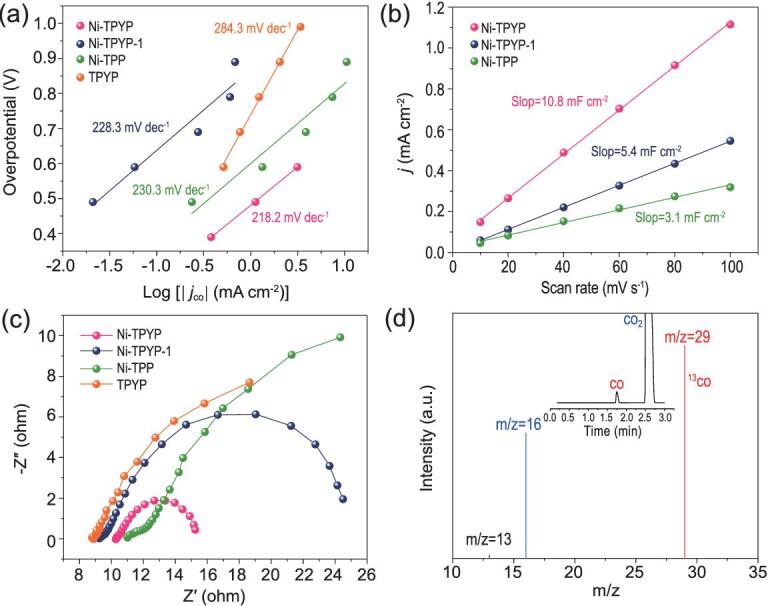
(a) Tafel plots of Ni-TPYP, Ni-TPYP-1, Ni-TPP and TPYP. (b) *C*_dl_ of Ni-TPYP, Ni-TPYP-1 and Ni-TPP. (c) Nyquist plots of electrocatalysts over the frequency ranging from 1000 kHz to 0.1 Hz at −0.9 V vs. RHE. (d) The mass spectra of ^13^CO recorded under ^13^CO_2_ atmosphere.

CO_2_RR test was carried out in aqueous solution of 0.5 M KHCO_3_ (pH = 7.2). CO is the main gaseous product detected by gas chromatography (Figs S15–S18). The ^1^H nuclear magnetic resonance (NMR) spectra show that no liquid product is observed (Fig. S19). The FE_CO_ of Ni-TPYP gradually increases in the potential range (−0.50 to −0.90 V vs. reversible hydrogen electrode (RHE)), and exhibits the highest FE_CO_ (99.8%) at −0.90 V. Interestingly, the FE_CO_ can maintain over 90% in a wide range of potential from −0.70 to −1.20 V (Fig. 2b and Fig. S20). Excellent performance compels us to explore its reaction mechanism with CO_2_RR. It has been reported that central metal in porphyrin is a catalytically active site for electrochemical CO_2_ reduction to CO. Therefore, for comparison, TPYP ligand was replaced by TPP to synthesize another very similar supramolecular structure (Ni-TPP). However, the corresponding electrochemical test results show that the CO_2_RR performance of Ni-TPP is much poorer than that of Ni-TPYP. As we can see from the linear scanning voltammetry (LSV) comparison curves (Fig. 2a), the total current density of Ni-TPP is lower than Ni-TPYP. Moreover, the highest FE_CO_ of Ni-TPP is only 25.6% at −0.90 V (Fig. 2c and Fig. S24), while the FE_CO_ of Ni-TPYP can reach up to 99.8% (Fig. 2c). Besides, the *j*_CO_ of Ni-TPYP is 10.7 mA cm^−2^ at −0.90 V, which is 18 times larger than that of Ni-TPP (0.6 mA cm^−2^) (Fig. 2d). Tafel slope for Ni-TPYP (218.2 mV dec^−1^) is also smaller than that of Ni-TPP (230.3 mV dec^−1^), implying the favorable kinetic for the CO formation [56], which is ascribed to the more efficient charge transfer and larger active surface in the catalytic process (Fig. 3a). To support this surmise, the electrochemical active surface areas (ECSA) for catalysts were estimated based on the double layer capacitive (*C*_dl_) by cyclic voltammetry at different scan rates. The result shows that the *C*_dl_ of Ni-TPYP (10.8 mF cm^−2^) is indeed larger than that of Ni-TPP (3.1 mF cm^−2^), which means that Ni-TPYP has a higher reaction speed in CO_2_RR process and more active sites that can have contact with the electrolyte (Fig. 3b and Fig. S25) [57]. Therefore, the CO_2_RR activity of Ni-TPYP is more active than Ni-TPP. Additionally, the turnover frequency (TOF) of Ni-TPYP is calculated to be 167.9 h^−1^ at −0.90 V, and achieves the maximum value (602.7 h^−1^) at −1.30 V (Fig. S26). By contrast, the TOF of Ni-TPP is only 7.6 h^−1^ at −0.90 V. In addition, an electrochemical impedance spectroscopy (EIS) measurement was carried out to probe the electrocatalytic kinetics on the electrode/electrolyte surface. Figure 3c demonstrates that Ni-TPYP has much smaller charge transfer resistance than Ni-TPP during the CO_2_RR process, indicating Ni-TPYP can transfer electrons from catalysts to reactants more quickly, eventually resulting in largely enhanced activity and selectivity. All these results indicate that Ni-TPYP has a better CO_2_RR performance than that of Ni-TPP, indicating that the activity of Ni is poor as a catalytic site. Given the relatively inert electrochemical activity of metal Ni, we infer that there must be other main factors (such as ligand effect) besides Ni for the excellent electroreduction activity of Ni-TPYP.

To gain further insight into the origin of activity of Ni-TPYP, we tested the CO_2_RR properties of pure ligands (TPYP and TPP). It clearly shows that the free TPYP ligand demonstrates poor catalytic performance with a maximum value of FE_CO_ only up to 10.1%. Hydrogen (H_2_) is the main product (Fig. S27). Besides, its total current density and *j*_co_ are all much smaller than that of Ni-TPYP (Fig. 2a–d and Fig. 3a). The CO_2_RR activities of free TPP ligand are also poorer than that of Ni-TPP, as demonstrated by its current density and FE_CO_ (Fig. S28 and Fig. S31). These results highlight the important role of metal Ni coordination. Once the metal ion Ni was trapped into the porphyrin center (Ni-TPYP), the electrocatalytic performance underwent tremendous changes. Thus, the metal coordination effect should be an indispensable factor responsible for the enhanced electroactivity of Ni-TPYP. Moreover, we found that the total current density of Ni-TPYP is larger than free TPYP (Fig. 2a). So, we inferred that in addition to acting as an active site, the Ni coordination is more likely to enhance the conductivity of the supramolecular structure, which is the main reason for the improved electrocatalytic performance of Ni-TPYP. EIS measurement further validates this fact, because Ni-TPYP has much smaller charge transfer resistance than TPYP (Fig. 3c and Fig. S32), which also exists between Ni-TPP and TPP (Fig. S33).

But beyond that, in the case of pure ligand (TPYP/TPP), the FE_CO_ of TPYP (10.1%) is higher than that of TPP (2.6%), which inspires us to speculate that the increase in electroreduction activity for pure ligands is attributed to the modification of active pyridine groups on porphyrin. Moreover, the positive role of active pyridine N in the CO_2_RR process has recently been demonstrated in some homogeneous catalysis [58] and nanostructured electrocatalysts (involving N-doped and N-heterocyclic nanomaterials) [31–35], in which pyridine N is considered as the superior catalytically active site and contributes to stabilization of the key intermediate to improve the performance of CO_2_RR. These results well coincide with our experimental results. However, they still lack sufficiently clear structure information. According to the above crystallographic and experimental results, our crystalline supramolecular coordination compounds can structurally prove that active pyridine N is indeed an important catalytic site for CO_2_RR.

According to the previous studies, in which pyridine was directly used as homogeneous catalyst for carbon dioxide reduction, methanol is the main product [58–60]. The electrolyte after electrolysis was further detected by headspace injection on gas chromatography. The test result is consistent with the ^1^H NMR test result mentioned above, i.e. there is no liquid product such as methanol (Fig. S34). By contrast, we also tested the CO_2_RR performance of pyridine under our experimental conditions. The test results are as shown in Fig. S35, there is no peak corresponding to methanol, which indicates that under our test conditions, pyridine will not reduce CO_2_ to methanol when used for CO_2_RR. The reason for this phenomenon should be related to the working electrode material. As previously reported, the reduction of pyridine does not occur at all electrode surfaces, because there is a critical surface interaction between pyridine and the working electrode related to pyridine reduction [61].

To further support our speculation that active pyridine N is a more suitable catalytic site, supramolecular coordination compound Ni-TPYP-1 was designed and synthesized, in which the Ni sites are saturated with six N atoms from TPYP ligands and two pyridine N atoms are naked. Thus, the attraction for CO_2_ from the metals in porphyrin centers is blocked by their saturated coordination sphere. As a result, CO_2_ can be only adsorbed by two naked pyridine N. The highest FE_CO_ of Ni-TPYP-1 can reach up to 83.7% at −0.90 V (Fig. 2b and c, and Fig. S36) and the *j*_CO_ of Ni-TPYP-1 is 7.4 mA cm^−2^ at −0.90 V vs. RHE (Fig. 2d). Tafel slope for Ni-TPYP-1 is 228.3 mV dec^−1^ (Fig. 3a). The *C*_dl_ of Ni-TPYP-1 is 5.4 mF cm^−2^ (Fig. 3b). All data indicate that Ni-TPYP-1 with two naked pyridine groups has poorer CO_2_RR performance than Ni-TPYP with four naked pyridine groups but much better than Ni-TPP without pyridine groups, which further confirms that active pyridine N is a better catalytic site.

In addition, another two isostructural complexes, Co-TPYP-1 and Zn-TPYP-1, which have the same host structure as Ni-TPYP-1, were further synthesized to confirm the accuracy of the above conclusions [62,63]. All the metal centers in the porphyrin units of Co-TPYP-1 and Zn-TPYP-1 are saturated with six N atoms, resulting in only two of the four available pyridyl arms of each porphyrin being naked (Figs S37–S40). The CO_2_RR test results show that the electroreduction performance of Co-TPYP-1 is also favorable. At relatively low potential (−0.50 V), its FE_CO_ is 63.0%, and then reaches up to the highest FE_CO_ (93.7%) at −0.70 V, which is a little better than Ni-TPYP-1 (Fig. S41). By contrast, the CO_2_RR performance of Zn-TPYP-1 is unsatisfactory. The highest FE_CO_ of Zn-TPYP-1 appears at –1.0 V with a value of 17.4% (Fig. S42). The difference in CO_2_RR performance is related to the type of metal trapped in porphyrin pocket. Different metal coordination effects endow metalloporphyrin complexes with different conductivity, and thus the differences in CO_2_RR performance. The results of EIS tests are consistent with our speculation, in which the metalporphyrin conductivity plays an important role in our system and there is such a sequence: Co-TPYP-1 > Ni-TPYP-1 > Zn-TPYP-1 (Fig. S43).

The excellent electrocatalytic performance of Ni-TPYP for CO formation encourages us to evaluate its durability. A 2-h electrolysis performed with a chronoamperometric test was carried out at −0.90 V in CO_2_-saturated KHCO_3_ aqueous solution. As time goes on, FE_CO_ continues to decline within 2 h and the activity remains above 80% in the previous hour (Fig. S44). The reaction liquids were collected and characterized by inductively coupled plasma mass spectrometry (ICP-MS) measurements and UV-visible spectrophotometer (Table S1 and Fig. S45). The content of metal element (Ni) in electrolyte after reaction is lower than the detection limit and there is no characteristic absorption peak of porphyrin in the ultraviolet-visible spectra, proving that no detectable impurities exist in the reaction electrolyte. In addition, the PXRD peaks of the samples before and after the electrochemical test were almost identical (Figs S46–S48). All these results further confirm the structural stability of electrocatalysts.

To verify the carbon source of the produced carbon monoxide, the isotopic ^13^CO_2_ experiment was performed under identical electrocatalytic reaction, and the product was identified by the mass spectrum of ^13^C. As shown in Fig. 3d, the mass spectrogram clearly gives the ^13^C signal, which is in line with previous work [47]. These results unambiguously demonstrate that Ni-TPYP is indeed active for converting CO_2_ to CO under electrolytic condition.

For most catalytic reactions (including CO_2_RR), the catalytic activity of heterogeneous catalysts mainly originates from surface/interface catalysis, which may affect the drawing of meaningful structure-property relationships, because in some cases, the structure of the surface may be different from the bulk crystal structure. Thus, we prepared Ni-TPYP microcrystal powder (as shown in Fig. S50 and Fig. S51, the size is ∼2 μm that is similar to the bulk crystal after grounding) through improved synthesis methods. The well-matched PXRD patterns for bulk crystals and microcrystal powder prove the purity of the synthesized microcrystal powder. Then, we used unground microcrystal powder of Ni-TPYP to prepare the electrode and tested its electrocatalytic CO_2_RR performance. We found that under identical electrocatalytic conditions, the main product of unground Ni-TPYP microcrystals is still CO, and FE_CO_ can still reach up to 98.41% (Fig. S52), further demonstrating the reliability of our experimental results and conclusions.

**Figure 4.**
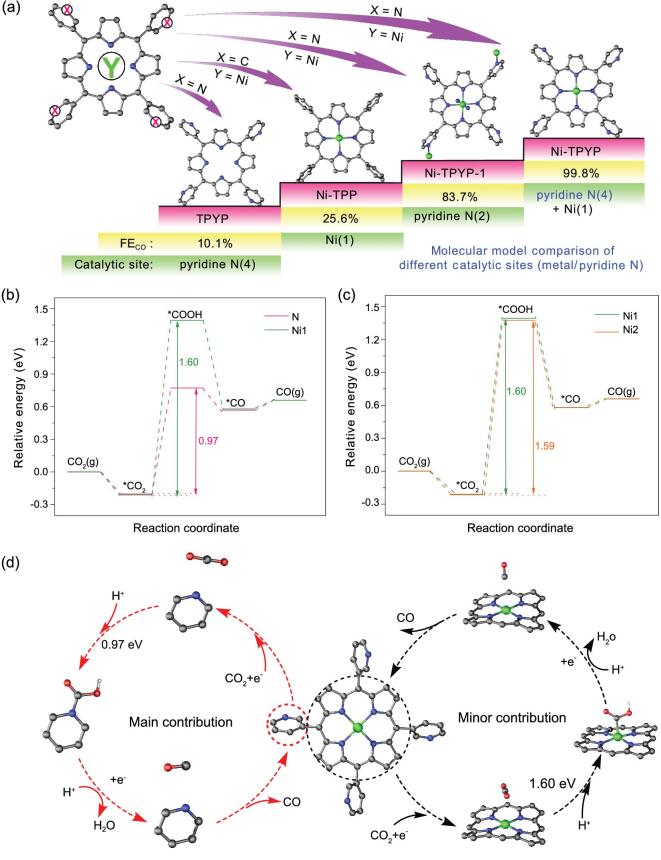
(a) Comparison of different molecular structures and catalytic sites (Ni-TPYP, Ni-TPYP-1, Ni-TPP and TPYP). (b, c) Calculated free energy profile for CO_2_RR toward the production of CO. (d) Simulated CO_2_-to-CO conversion reactive pathway over Ni-TPYP molecule.

According to the above experimental results, by controlling the catalytically active sites, the CO_2_RR activity of pyridine N can be clearly confirmed, as shown in Fig. 4a. When a central four-coordination metal (Ni) and four pyridine groups are naked, the supramolecular compound Ni-TPYP has the highest FE_CO_ (99.8%), while when four pyridine groups are replaced by benzenes (Ni-TPP), the catalytic site at this time is only the metal Ni. The electrochemical test results show that the activity of Ni-TPP is poor, and the maximum value of FE_CO_ is only 25.6%, indicating that Ni is not the best active site. The fact that the CO_2_RR performance of TPYP is better than that of TPP inspired us to speculate that the excellent properties of Ni-TPYP maybe mainly due to the pyridine groups modified on porphyrin. Furthermore, the compound Ni-TPYP-1 was synthesized to further demonstrate that pyridine N can act as catalytic active site. The metal Ni of Ni-TPYP-1 is saturated six coordination and two pyridine groups are naked which can act as catalytically active sites. The test results show that the CO_2_RR performance of Ni-TPYP-1 is poorer than that of Ni-TPYP but much better than that of Ni-TPP, which further proves the above speculation that active pyridine N is the more suitable catalytic site. However, it is noteworthy that the role of metal Ni cannot be ignored; it is more likely to enhance the conductivity of molecular structure by coordination effect in addition to acting as a catalytic site.

Density functional theory (DFT) calculation was carried out to further identify the catalytically active sites in these crystalline electrocatalysts. Generally, four elementary reactions were considered during the CO_2_RR process, including the formation of ^*^CO_2_, ^*^COOH, ^*^CO and finally the CO desorption processes. Two kinds of active sites (metal center and pyridine N) were taken into account in our calculations. From the DFT calculation diagrams shown in Fig. 4a and b, the RDS for CO_2_ reduction is the formation of ^*^COOH. In the case of the metal active site, the active Ni in Ni-TPYP (denoted as Ni1) requires free energy of 1.60 eV to form intermediate ^*^COOH, which is almost the same as that of active Ni in Ni-TPP (denoted as Ni2, 1.59 eV). This result indicates that the activity of Ni1 and Ni2 is similar, and the high energy barriers of ^*^COOH formation show that Ni2 possesses poor activity, and so does Ni1. Interestingly, for the active pyridine N site in Ni-TPYP (denoted as N), it consumes the lowest energy difference of 0.97 eV to form ^*^COOH. The reduction processes from CO_2_ to CO on Ni-TPYP are summarized in Fig. 4c. All in all, the sequence of the reaction energies on these two active sites reveals that the high CO_2_ electroreduction performance for Ni-TPYP is mainly attributed to the active pyridine N.

## CONCLUSION

In summary, by hierarchical controlling of the number of catalytic sites in supramolecular coordination compounds crystal model system, the catalytic activity of pyridine-containing materials was for the first time structurally identified in CO_2_RR. The system includes Ni-TPYP, Ni-TPYP-1 and Ni-TPP, which have four uncoordinated pyridine N, two uncoordinated pyridine N and no pyridine N atoms, respectively. As the number of uncoordinated pyridine N atoms decreases, the selectivity of the compounds exhibits such a tendency: Ni-TPYP (FE_CO _= 99.8%) > Ni-TPYP-1 (83.7%) > Ni-TPP (25.6%). These results clearly demonstrated that the electrocatalytic performance for Ni-TPYP mainly comes from the active pyridine N atom. Among them, the Ni coordination effect plays a very important role in improving the conductivity of the electrocatalyst. Theoretical calculations also show that CO_2_ reacts more readily with pyridine N than metal Ni. Significantly, such crystalline supramolecular coordination compounds crystal model system exactly identifies the activity of pyridine N in CO_2_RR process. This work provides a simple and accurate supramolecular coordination compounds model system to understand the electrocatalytic activity and reaction mechanism of N-doped and N-heterocyclic nanostructured electrocatalysts in CO_2_RR.

## METHODS

### Materials and synthetic procedures

Porphyrin ligands and methanol were obtained from commercial sources. Nickel acetate tetrahydrate (Ni(OAc)_2_·4H_2_O), cobalt acetate tetrahydrate (Co(OAc)_2_·4H_2_O), zinc nitrate hexahydrate (Zn(NO_3_)_2_·6H_2_O), potassium bicarbonate (KHCO_3_), pyridine (99+%), hydrochloric acid (HCl) and N,N-dimethylformamide (DMF) were supplied by the Shanghai Reagent Factory. Nafion solution (5 wt %) was purchased from Sigma–Aldrich. Carbon paper (CP, TGP-H-060) was bought from Fuel Cell Store. All of the chemicals used in this experiment were analytical grade and used without further purification. All aqueous solutions were prepared with Millipore water (18.20 MΩ·cm). The purity of the gases used was 99.999%.

### Synthesis of all samples

#### Synthesis of Ni-TPYP crystal

The 6 mL mixed solution of deionized water and DMF containing 5,10,15,20-tetra(3pyridiyl)-21H, 23H-porphine (0.06 g, 0.10 mmol, TPYP), and Ni(OAc)_2_·4H_2_O (0.06 g, 0.10 mmol) was placed into a 10 mL scintillation vial and heated at 150°C for 3 days. After cooling down to room temperature with a rate of 10°C h^−1^, the black octahedral crystal was collected and washed with DMF several times.

#### Synthesis of Ni-TPYP-1 crystal

The 6 mL mixed solution of deionized water, trimethylamine and DMF (2:1:3) containing TPYP (0.03 g, 0.05 mmol), and Ni(OAc)_2_·4H_2_O (0.03 g, 0.125 mmol) was placed into a 10 mL scintillation vial and heated at 180°C for 3 days. After cooling down to room temperature with a rate of 10°C h^−1^, the black diamond crystal was collected and washed with DMF several times.

#### Synthesis of Ni-TPP crystal

The 5,10,15,20-tetraphenyl-21H,23H-porphine (0.06 g, 0.10 mmol, TPP) was added to the mixed solution (6 mL) of DMF and deionized water. Then triethylamine (28 μL, 0.20 mmol) was added to the above solution. After it was sonicated for 30 min, Ni(OAc)_2_·4H_2_O (0.06 g, 0.10 mmol) was added into the mixed solution and sonicated for another 30 min. The solution was placed in a 10 mL scintillation vial and heated at 150°C for 72 h, and the black octahedral crystal was obtained.

#### Synthesis of Co-TPYP-1 crystal

The mixed solution of 4 mL DMF and 41.61 μL HCl containing TPYP (0.0062 g, 0.01 mmol) and Co(OAc)_2_·4H_2_O (0.005 g, 0.02 mmol) was placed into a 10 mL scintillation vial and heated at 150°C for 2 days. After cooling down to room temperature with a rate of 10°C h^−1^, the black diamond crystal was collected and washed with DMF several times.

#### Synthesis of Zn-TPYP-1 crystal

The 4 mL DMF containing TPYP (6.2 mg, 0.01 mmol), and Zn(NO_3_)_2_·6H_2_O (6.0 mg, 0.02 mmol) was placed into a small capped vial. The vial contents were mixed by sonication and heated at 100°C for 25 h. After cooling down to room temperature over 35 h, the purple diamond crystal was collected and washed with DMF several times.

#### Synthesis of Ni-TPYP microcrystal powder

The 0.06 g (0.10 mmol) 5,10,15,20-tetra (3pyridiyl)-21H, 23H-porphine (TPYP) and 0.06 g (0.10 mmol) Ni(OAc)_2_·4H_2_O were placed into a 50 mL flask. Then 25 mL DMF was added into the flask. The mixture was refluxed for 10 h. When the reaction was completed, a lot of water was added to precipitate the product. Reddish brown powder (Ni-TPYP microcrystal powder) was collected and washed with H_2_O several times.

### Electrochemical measurements

All electrochemical tests of the catalysts were performed on an electrochemical workstation (Bio-Logic) with a standard three-electrode system at room temperature and under ambient conditions. An airtight H-type cell separated by a cation exchange membrane (Nafion®117, dupont) was used as a reactor. Each compartment contained 25 mL electrolyte (0.5 M KHCO_3_) with approximately 25 mL headspace. An Ag/AgCl electrode (with saturated KCl) and a carbon rod were used as the reference electrode and the counter, respectively. The working electrode was a catalyst-modified carbon paper (1 cm × 2 cm, denoted as CPE) in 0.5 M KHCO_3_ solution (pH = 7.2).

The polarization curves results were obtained by performing linear sweep voltammetry (LSV) mode with a scan rate of 5 mV s^−1^ during the CO_2_ reduction experiments. Initially, LSV for the modified electrode was recorded in Ar-saturated 0.5 M KHCO_3_ (pH = 8.8). The LSV result in Ar-saturated 0.5 M KHCO_3_ was achieved after bubbling with CO_2_ (99.999%) for at least 30 min to make sure the aqueous solution was saturated. All the LSV curves were presented without IR compensation. After that, the controlled potential electrolysis was conducted. All potentials were measured against an Ag/AgCl reference electrode and the results were converted to those against an RHE based on the Nernst equation: E (vs. RHE) = E (vs. Ag/AgCl) + 0.1989 V + 0.059 × pH.

ECSA measurement was estimated by performing cyclic voltammograms (CV) at different scan rates from 10 to 100 mV s^−1^ under the potential window of −0.18 V to −0.06 V (vs. RHE) to measure the double-layer capacitance (*C*_dl_).

EIS measurement was carried out on the electrochemical analyzer in a frequency range from 1000 kHz to 100 mHz at an overpotential of −0.90 V vs. RHE.

## Supplementary Material

nwaa195_Supplemental_File
