# Supplementary material for: Identification of the activity source of CO2 electroreduction by strategic catalytic site distribution in stable supramolecular structure system
Source: Natl Sci Rev. 2020 Aug 31;8(3):nwaa195. doi: 10.1093/nsr/nwaa195 (PMC8288368; doi:10.1093/nsr/nwaa195)
Supplement: nwaa195_Supplemental_File [file nwaa195_supplemental_file.zip › Revised Supporting Information.doc]

Supporting Information

**Identification of the Activity Source of CO_2_ Electroreduction by Strategic Catalytic Site Distribution in Stable Supramolecular Structure System**

Sheng-Nan Sun,^1,†^ Ning Li, ^2,†^ Jiang Liu,*^1^ Wen-Xin Ji,^3^ Long-Zhang Dong,^1^ Yi-Rong Wang, ^1^ and Ya-Qian Lan*^1^

*^1^ Jiangsu Collaborative Innovation Centre of Biomedical Functional Materials, Jiangsu Key Laboratory of New Power Batteries, School of Chemistry and Materials Science, Nanjing Normal University, Nanjing, 210023, P. R. China.*

*^2^ School of Chemistry and Chemical Engineering, Yangzhou University Yangzhou 225002, P. R. China.*

*^3^ State Key Laboratory of High-efficiency Coal Utilization and Green Chemical Engineering, Ningxia University, Yinchuan 750021, China.*

*^†^ These authors contributed equally*

**Corresponding authors* *J. L.* (email: liuj@njnu.edu.cn) and *Y.-Q. L.* (email: yqlan@njnu.edu.cn)

**Fig. S1** The image of Ni-TPYP under optical microscope. As shown in the figure, Ni-TPYP is black octahedron.

**Fig. S2** The image of Ni-TPP under optical microscope. As shown in the figure, Ni-TPP is black octahedron.

**Fig. S3** Structure of crystals with hydrogen bond. (a) Ni-TPYP. (b) Ni-TPP. Color code: C, black; N, blue; Ni, green.

**Fig. S4** The image of Ni-TPYP-1 under optical microscope. As shown in the figure, Ni-TPYP-1 is black diamond.

**Fig. S5** View along the b axis of the stacked structure of Ni-TPYP-1. Color code: C, black; N, blue; Ni, green. Each Ni^II^ ion trapped in TPYP center coordinates two N atoms of pyridine from another two TPYP ligands to form a 2D layer and the layers are further stacked into supramolecular coordination compounds by π-π interaction.

**Fig. S6** The structure images of ligands. (a) TPYP. (b) TPP. All ligands were purchased commercially and not further purified.

**Fig. S7** PXRD patterns of Ni-TPYP (2 Theta ranges from 5° to 50°). “Sim”: simulated pattern and “Exp”: as-synthesized sample. The PXRD of as-synthesized sample was collected by Powder X-Ray diffraction (PXRD) measurements ranging from 5 to 50° at room temperature.

**Fig. S8** PXRD patterns of Ni-TPYP-1 (2 Theta ranges from 5° to 50°). “Sim”: simulated pattern and “Exp”: as-synthesized sample. The PXRD of as-synthesized sample was collected by Powder X-Ray diffraction (PXRD) measurements ranging from 5 to 50° at room temperature.

**Fig. S9** PXRD patterns of Ni-TPP (2 Theta ranges from 5° to 50°). “Sim”: simulated pattern and “Exp”: as-synthesized sample. The PXRD of as-synthesized sample was collected by Powder X-Ray diffraction (PXRD) measurements ranging from 5 to 50° at room temperature.

**Fig. S10** PXRD patterns of Ni-TPYP (2 Theta ranges from 5° to 50°) in acid, base and 0.5 M KHCO_3_ solutions. The pH stability was investigated by immersing the samples of Ni-TPYP into different pH aqueous solutions and 0.5 M KHCO_3_ aqueous solution ranging from 3 to 13 for 24 hours.

**Fig. S11** PXRD patterns of Ni-TPYP-1 (2 Theta ranges from 5° to 50°) in acid, base and 0.5 M KHCO_3_ solutions. The pH stability was investigated by immersing the samples of Ni-TPYP into different pH aqueous solutions and 0.5 M KHCO_3_ aqueous solution ranging from 3 to 13 for 24 hours.

**Fig. S12** PXRD patterns of Ni-TPP (2 Theta ranges from 5° to 50°) in acid, base and 0.5 M KHCO_3_ solutions. The pH stability was investigated by immersing the samples of Ni-TPYP into different pH aqueous solutions and 0.5 M KHCO_3_ aqueous solution ranging from 3 to 13 for 24 hours.

**Fig. S13** FT-IR spectra of crystals of Ni-TPYP, Ni-TPYP-1 and Ni-TPP. Fourier transform infrared spectroscopy (FT-IR) was recorded on a Bruker Tensor 27 in the range of 4000-400 cm^-1^ using the technique of pressed KBr pellets.

**Fig. S14** The TGA curves of Ni-TPYP, Ni-TPYP-1 and Ni-TPP. The thermal stability of samples was studied by thermogravimetric analysis (TGA) under O_2_ atmosphere heated from room temperature to 700 °C, which revealed that all these two samples could preserve their structural integrity up to 400 °C. The high thermal stability of samples lay a foundation for further investigation of electroreduction performance and applications.

**Fig. S15** Gas chromatography of product (CO). The gase (CO) was detected and analyzed by GC (GC-7920) equipped with a flame ionization detector (FID) and a thermal conductivity (TCD). Among them, FID was used to analyze CO.

**Fig. S16** Gas chromatography of product (H_2_). The gase (H_2_) was detected and analyzed by GC (GC-7920) equipped with a flame ionization detector (FID) and a thermal conductivity (TCD). Among them, TCD was used to analyze H_2_.

**Fig. S17** Standard curve of product (CO). The gas used to make the standard curve is commercially available.

**Fig. S18** Standard curve of product (H_2_). The gas used to make the standard curve is commercially available.

**Fig. S19** Characterization for the liquid product of Ni-TPYP during CO_2_ reduction process by ^1^H nuclear magnetic resonance spectroscopy. There are only H_2_O and DMSO peaks in the figure, indicating that there is no product in the liquid phase.

**Fig. S20** Faradaic efficiencies (CO and H_2_) of Ni-TPYP at different applied potentials in CO_2_-saturated 0.5 M KHCO_3_ aqueous solution. The values of FE was calculated according to supplementary equation 1 in supplementary note 2.

**Fig. S21** Linear sweep voltammetric (LSV) curves of Ni-TPYP in Ar-saturated and CO_2_-saturated 0.5 M KHCO_3_ aqueous solution.

**Fig. S22** Linear sweep voltammetric (LSV) curves of Ni-TPYP-1 in Ar-saturated and CO_2_-saturated 0.5 M KHCO_3_ aqueous solution

**Fig. S23** Linear sweep voltammetric (LSV) curves of Ni-TPP in Ar-saturated and CO_2_-saturated 0.5 M KHCO_3_ aqueous solution.

**Fig. S24** Faradaic efficiencies (CO and H_2_) of Ni-TPP at different applied potentials in CO_2_-saturated 0.5 M KHCO_3_ aqueous solution. The values of FE was calculated according to supplementary equation 1 in supplementary note 2.

**Fig. S25** Cyclic voltammertrys (CV) curves in the region of -0.18 ~-0.06 V vs. RHE at various scan rate (10 ~ 100 mV s^-1^) and corresponding capacitive current at -0.12 V as a function of scan rate for (a), (b) Ni-TPYP. (c), (d) Ni-TPYP-1. (e), (f) Ni-TPP. Electrochemical active surface area (ECSA) measurement was estimated by CV at different scan rates from 10 to 100 mV s^-1^ under the potential window of -0.18 V to -0.06 V (vs. RHE) to measure the double-layer capacitance (C_dl_).

**Fig. S26** Plots of CO evolving turnover number versus time for Ni-TPYP, Ni-TPYP-1 and Ni-TPP. The values of TOF was calculated according to supplementary equation 3 in supplementary note 2.

**Fig. S27** Faradaic efficiencies (CO and H_2_) of TPYP at different applied potentials in CO_2_-saturated 0.5 M KHCO_3_ aqueous solution. The values of FE_CO_ was calculated according to supplementary equation 1 in supplementary note 2.

**Fig. S28** Faradaic efficiencies (CO and H_2_) of TPP at different applied potentials in CO_2_-saturated 0.5 M KHCO_3_ aqueous solution. The values of FE_CO_ was calculated according to supplementary equation 1 in supplementary note 2.

**Fig. S29** Faradaic efficiencies (H_2_) comparison plots of Ni-TPYP, Ni-TPYP-1, Ni-TPP and TPYP in CO_2_-saturated 0.5 M KHCO_3_ aqueous solution. The values of FE_CO_ was calculated according to supplementary equation 1 in supplementary note 2.

**Fig. S30** Partial H_2_ current density (based on geometric surface area) plots of Ni-TPYP, Ni-TPYP-1, Ni-TPP and TPYP in CO_2_-saturated 0.5 M KHCO_3_ aqueous solution. The values of FE_H2_ were calculated according to supplementary equation 2 in supplementary note 2.

**Fig. S31** LSVs of Ni-TPYP, Ni-TPYP-1, Ni-TPP, TPYP and TPP in CO_2_-saturated 0.5 M KHCO_3_ solution.

**Fig. S32** Nyquist plots of Ni-TPYP and TPYP over the frequency ranging from 1000 kHz to 0.1 Hz at -0.90 V vs. RHE. EIS measurements show that Ni-TPYP has much smaller charge transfer resistance than TPYP, indicating the Ni coordination is more likely to enhance the conductivity of supramolecular structure.

**Fig. S33** Nyquist plots of Ni-TPP and TPP over the frequency ranging from 1000 kHz to 0.1 Hz at -0.90 V vs. RHE. EIS measurements show that Ni-TPP has much smaller charge transfer resistance than TPP, indicating the Ni coordination is more likely to enhance the conductivity of supramolecular structure.

**Fig. S34** The chromatogram of the electrolyte solution from the cathode chamber after electrolysis (black line) and 10ppm methanol solution (red line). The volatile liquid products in solution were detected by headspace injection. Detector is flame ionization detector (FID). There is no peak of methanol on the black line, indicating that there is no methanol in the liquid phase after electrolysis. Small peaks other than methanol are impurities in chromatographic column or drugs.

**Fig. S35** The chromatogram of the electrolyte solution from the cathode chamber after electrolysis (black line) and 10ppm methanol solution (red line). The volatile liquid products in solution were detected by headspace injection. Detector is flame ionization detector (FID). Small peaks other than methanol are impurities in chromatographic column or drugs.

The contrast experiment was conducted under our experimental conditions. An airtight H-type cell separated by a cation exchange membrane (Nafion®117, dupont) was used as a reactor. Each compartment contained 25 mL electrolyte (0.5 M KHCO_3_) containing 10 mM pyridine with approximately 25 mL headspace. Ag/AgCl electrode (with saturated KCl) and carbon rod were used as the reference electrode and the counter, respectively. The working electrode was a carbon paper (1 cm × 2 cm). There is no peak of methanol on the black line, indicating that under our test conditions, pyridine cannot reduce CO_2_ to methanol.

**Fig. S36** Faradaic efficiencies (CO and H_2_) of Ni-TPYP-1 at different applied potentials in CO_2_-saturated 0.5 M KHCO_3_ aqueous solution. The values of FE was calculated according to supplementary equation 1 in supplementary note 2.

**Fig. S37** The stacked structure of Co-TPYP-1. Color code: C, black; N, blue; Co, pink. Each Co^II^ ion trapped in TPYP center coordinates two N atoms of pyridine from another two TPYP ligands to form a 2D layer and the layers are further stacked into supramolecular coordination compounds by π-π interaction.

**Fig. S38** The stacked structure of Zn-TPYP-1. Color code: C, black; N, blue; Zn, yellow. Each Zn^II^ ion trapped in TPYP center coordinates two N atoms of pyridine from another two TPYP ligands to form a 2D layer and the layers are further stacked into supramolecular coordination compounds by π-π interaction.

**Fig. S39** PXRD patterns of Co-TPYP-1 (2 Theta ranges from 5° to 50°). “Sim”: simulated pattern and “Exp”: as-synthesized sample.

**Fig. S40** PXRD patterns of Zn-TPYP-1 (2 Theta ranges from 5° to 50°). “Sim”: simulated pattern and “Exp”: as-synthesized sample.

**Fig. S41** Faradaic efficiencies (CO and H_2_) of Co-TPYP-1 at different applied potentials in CO_2_-saturated 0.5 M KHCO_3_ aqueous solution. The values of FE was calculated according to supplementary equation 1 in supplementary note 2.

**Fig. S42** Faradaic efficiencies (CO and H_2_) of Zn-TPYP-1 at different applied potentials in CO_2_-saturated 0.5 M KHCO_3_ aqueous solution. The values of FE was calculated according to supplementary equation 1 in supplementary note 2.

**Fig. S43** Nyquist plots of Co-TPYP-1, Ni-TPYP-1 and Zn-TPYP-1 over the frequency ranging from 1000 kHz to 0.1 Hz at -0.90 V vs. RHE. EIS measurements show that Co-TPYP-1 has much smaller charge transfer resistance than Ni-TPYP-1 and Zn-TPYP-1, indicating the conductivity of Co-TPYP-1 is the best, yet Zn-TPYP-1 is the worst.

**Fig. S44** Stability of Faradaic efficiency of Ni-TPYP for CO_2_ reduction at the potentiostatic mode of -0.9V vs. RHE. As time goes on, FE_CO_ continues to decline within two hours and the activity remained above 80% in the previous hour. Besides, the decay of current may be due to the decrease of catalyst activity.

**Fig. S45** Spectrogram of Ultraviolet Spectrophotometer of tested electrolyte. After electrolysis, reaction liquids were collected and characterized by UV-visible spectrophotometer (red line). There is no characteristic absorption peak in the ultraviolet-visible spectra, proving that no detectable impurities exist in the reaction electrolyte. The porphyrin ligand was immersed in the electrolyte solution. After ultrasonic treatment for 1 hour, the supernatant was taken for liquid UV test (blue line). Because the porphyrin ligand is hydrophobic, so no ligand is dissolved in the electrolyte.

**Fig. S46** PXRD patterns of Ni-TPYP before and after test. The PXRD pattern of Ni-TPYP after electrochemical experiment matches well with the simulated and as-synthesized samples, confirming the structural stability of electrocatalyst.

**Fig. S47** PXRD patterns of Ni-TPYP-1 before and after test. The PXRD pattern of Ni-TPYP-1 after electrochemical experiment matches well with the simulated and as-synthesized samples, confirming the structural stability of electrocatalyst.

**Fig. S48** PXRD patterns of Ni-TPP before and after test. The PXRD pattern of Ni-TPP after electrochemical experiment matches well with the simulated and as-synthesized samples, confirming the structural stability of electrocatalyst.

**Fig. S49** Faradaic efficiencies (CO and H_2_) of pure carbon paper at different applied potentials in CO_2_-saturated 0.5 M KHCO_3_ aqueous solution. The bare carbon paper was measured adopting the same testing methods to conduct its CO_2_RR activity. As shown in the image, pure carbon paper has no CO_2_RR activity.

**Fig. S50** (a) The images of Ni-TPYP bulk crystal under optical microscope. (b) The SEM of Ni-TPYP after grinding by a ball mill for 30 minutes.

**Fig. S51** (a) PXRD patterns of Ni-TPYP microcrystal powder. “Sim”: simulated pattern. (b) SEM image of Ni-TPYP microcrystal powder. (c) The diameter distribution of Ni-TPYP microcrystal powder (the diameter is the longest distance between two vertices and calculated based on the Nano-measurer software). (d) TEM image of Ni-TPYP microcrystal powder.

**Fig. S52** Faradaic efficiencies (CO and H_2_) of Ni-TPYP microcrystal powder at different applied potentials in CO_2_-saturated 0.5 M KHCO_3_ aqueous solution.

**Table S1.** ICP data of tested electrolyte of Ni-TPYP.

| **Instrument** | **Element** | **Content (mg/L)** |
| --- | --- | --- |
| ICP | Ni | < detection limit |
| ICP-MS | Ni | < detection limit |

After electrolysis, reaction liquids were collected and characterized by inductively coupled plasma mass spectrometry (ICP-MS) measurements. The content of metal element (Ni) in electrolyte after reaction is lower than the detection limit, proving that no detectable impurities exist in the reaction electrolyte.

**Table S2.** Crystal data and structure refinement for Ni-TPYP, Ni-TPYP-1 and Ni-TPP.

| **Compounds** | **Ni-TPYP** | **Ni-TPYP-1** | **Ni-TPP** |
| --- | --- | --- | --- |
| **Empirical formula** | C_40_H_24_N_8_Ni | C_40_H_24_N_8_Ni | C_44_H_28_N_4_Ni |
| **Formula weight** | 675.38 | 675.38 | 671.41 |
| **Crystal system** | Tetragonal | Monoclinic | Tetragonal |
| **Space group** | *I*-42*d* | *P*2_1_*/c* | *I*-42*d* |
| ***a* (Å)** | 16.783(4) | 11.720(16) | 15.23(2) |
| ***b* (Å)** | 16.783(4) | 9.837(13) | 15.23(2) |
| **c (Å)** | 11.042(3) | 14.003(19) | 14.11(2) |
| ***α* (°)** | 90 | 90 | 90 |
| ***β* (°)** | 90 | 110.324(17) | 90 |
| ***γ* (°)** | 90 | 90 | 90 |
| ***V* (Å^3^)** | 3110.1(16) | 1514(3) | 3271(12) |
| ***Z*** | 4 | 2 | 4 |
| ***D*_calc_(Mg·m^-3^)** | 1.442 | 1.482 | 1.363 |
| **Abs.coeff.(mm^-1^)** | 0.669 | 0.687 | 0.632 |
| ***F*(000)** | 1392.0 | 696.0 | 1392.0 |
| **Reflns collected** | 19846 | 12741 | 6951 |
| **Independent reflns** | 1774 | 2688 | 1593 |
| **GOF** **on *F*^2^** | 1.073 | 1.018 | 1.029 |
| ***R*_int_** | 0.0255 | 0.1077 | 0.0602 |
| ***R_1_* [*I* > 2*σ*(*I*)]^a^** | 0.0232 | 0.0633 | 0.0777 |
| ***wR_2_* [*I* > 2*σ*(*I*)]^a^** | 0.0597 | 0.1181 | 0.2153 |
| ***R*_1_(all data)^b^** | 0.0249 | 0.1286 | 0.0995 |
| ***wR*_2_(all data)^b^** | 0.0606 | 0.1456 | 0.2381 |

^a^ *R*1 =Σ||*F_o_*| – |*F_c_*||/Σ|*F_o_*|. ^b^ *wR*_2_ = |Σw(|*F_o_*|^2^ – |*F_c_*|^2^)|/Σ|w(*F_o_*^2^)^2^|^1/2^.

**Supplementary Note 1: Characterizations and instruments**

Powder X-Ray diffraction (PXRD) measurements of the samples were performed on a D/max 2500 VL/PC diffractometer (Japan) equipped with Cu Kα (λ = 1.54060 Å) radiation from the 2θ = 5 to 50° at room temperature. Fourier transform infrared spectroscopy (FT-IR) was recorded on a Bruker Tensor 27 in the range of 4000-400 cm^-1^ using the technique of pressed KBr pellets. Thermogravimetric analysis (TGA) of crystal samples was performed on a Netzsch STA449F3 analyzer under the oxygen atmosphere heated from room temperature to 700 °C with a heating rate of 10 °C min^-1^. The UV-Vis absorption spectra was acquired on a Shimadzu UV-2550 spectrophotometer in the wavelength range of 200–800 nm. Inductively coupled plasma mass spectrometry (ICP-MS) measurement was performed with Agilent-720. The isotope-labeled experiment was performed using ^13^CO_2_ instead of ^12^CO_2_. Single-crystal XRD datas of Ni-TPYP, Ni-TPYP-1 and Ni-TPP were measured on a Bruker APEXII CCD diffractometer with graphite-monochromated Mo Kα radiation (λ = 0.71073 Å) at 296 K. All the structures were solved by direct method using SHELXT program and refined on an Olex2 software. The detailed crystallographic information of these crystals was listed in Table S2. The CCDC number of Ni-TPYP, Ni-TPYP-1 and Ni-TPP are 1939400, 1949160 and 1939399.

**Supplementary Note 2: The preparation of the CPE working electrode.**

When preparing the woking electrode, acetylene black (AB) was incorporated into the as-synthesized single molecule material to improve the conductivity. In order to better attach the catalyst to the surface of carbon paper, it is necessary to introduce Nafion solution to catalyst, which can form a homogeneous ink with materials and generally applied in many reported works. The carbon paper was used as the working electrode substrate. The pure carbon paper has no CO_2_RR activity (Fig. S49). To put it simply, 10 mg of catalyst were mixed with 10 mg of AB, and then the mixture was dispersed in 1 mL of 0.5wt% Nafion solution. After sonicating for 30 min to form homogeneous ink, the ink was directly dropped onto a carbon paper (1 cm × 2 cm) to form a 1×1 cm^2^ catalyst area with a catalyst loading density of ~1 mg cm^-2^.

**Supplementary Note 3: Analysis of CO_2_ reduction product**

The bulk electrolysis was carried out in an airtight electrochemical H-type cell at selected potentials to determine the reduction products and their Faradic efficiency. The gaseous reduction products (e.g., CO) were monitored by a gas chromatography (GC-7920) equipped with a flame ionization detector (FID). During the test, helium was the carrier gas. A thermal conductivity detector (TCD) was used to analyze hydrogen with nitrogen as the carrier gas. The electrolyte solution was collected from the cathode chambers after electrolysis and characterized by ^1^H nuclear magnetic resonance (^1^H NMR), which was recorded with a Bruker 500 MHz NMR instrument. The ^1^H NMR spectra shows that no liquid product is observed, in which 0.5 mL electrolyte was mixed with 0.1 mL D_2_O and 0.1 μL dimethyl sulfoxide (DMSO, 99.99%, internal standard). Solvent pre-saturation technique was implemented to suppress the water peak.

**Supplementary Note 4: The relevant calculation equations**

The calculation of Faradaic efficiency:

FE =
 100% (Supplementary Equation 1)

where *Z* is the number of electrons exchanged (for example, *Z* = 2 for reduction of CO_2_ to CO), *F* is the Faraday constant (*F* = 96485 C mol^-1^ ), is the moles of a specific product_,_ *Q* is the charge passed (C).

Partial current density (*j*_product_) of each product was calculated as follows:

*j*_product_ = *j*_total_ × FE_product_ (Supplementary Equation 2)

where *j*_total_ is the average total current density (mA cm^-2^) during electrolysis.

Turnover Frequency (TOF, h^-1^)

The TOF for CO was calculated as follow:

TOF = (Supplementary Equation 3)

 : partial current for specific product;

N: the number of electron transferred for product formation, which is 2 for CO;

*F*: Faradaic constant, 96485 C mol^-1^;

: catalyst mass in the electrode, g;

ω: catalytic site loading in the catalyst;

: atomic mass of catalytic site.

**Supplementary Note 5: Density functional theory (DFT) calculation**

The calculations were performed using the ORCA package employing the resolution of identity approximation [10]. All the DFT calculations were performed using the hybrid B3LYP functional. Basis sets of def2-TZVP [11] were adopted for all atoms in the complexes with decontracted auxiliary def2-TZVP/J Coulomb fitting basis sets [12-13]. Increased integration grids (“Grid4”in ORCA convention) and tight SCF convergence criteria were used throughout. D3 dispersion correction developed by Grimme is included for weak interactions [14]. The solvation effects in aqueous solution have been considered by using the Conductor-like Continuum Polarization Model [15], the dielectric constant is taken as 80.4 for water.

The computational hydrogen electrode (CHE) model that proposed by Nørskov et al. [16] was applied to describe the Gibbs reaction free energy of reaction for CO_2_RR elementary steps involving (H^+^ + e^–^) pair transfer. The calculation for each step can be defined as Δ*G_n_*(U) = Δ*G_n_*(U=0) + *n*e*U*, where e is the elementary charge of an electron, *n* is the number of (H^+^ + e^–^) pairs transferred in CO_2_RR and *U* is the electrode potential versus the reversible hydrogen electrode (RHE). Specifically, Δ*G* = Δ*E* + Δ*E*_ZPE_ − *T*Δ*S* + ∫*C_P_*d*T* at *U*=0 V,where Δ*E* is the reaction energy difference between the product and reactant of the CO_2_RR occurring on catalysts, which can be directly obtained from DFT computations; Δ*E*_ZPE_, *T*Δ*S* and ∫*Cp*d*T* are zero-point energy correction, entropy correction and enthalpic temperature correction at T=298.15 K respectively, which were calculated from the vibrational frequencies. The Δ*E*_ZPE_, *T*Δ*S* and ∫*Cp*d*T* for each reaction intermediates can be calculated by the following equations, respectively:

| $E_{ZPE}=\frac{1}{2}\sum_{i} hv_{i}$ | （1） |
| --- | --- |
| $-TS=K_{B}T\sum_{i} \ln(1-e^{-\frac{hv_{i}}{K_{B}T}})-\sum_{i} hv_{i}(\frac{1}{e^{\frac{hv_{i}}{K_{B}T}}-1})$ | （2） |
| $\int C_{p}dT=\sum_{i} hv_{i}(\frac{1}{e^{\frac{hv_{i}}{K_{B}T}}-1})$ | （3） |

where *h*, *v* and *K*B are Planck constant, vibrational frequencies and Boltzmann constant, respectively.

**Reference:**

1.	Zhu D D and Liu J LQiao S Z. Recent advances in inorganic heterogeneous electrocatalysts for reduction of carbon dioxide. *Adv. Mater.* 2016; **28:** 3423-3452.

2.	Wang X, Chen Z, Zhao X *et al.* Regulation of coordination number over single Co sites: triggering the efficient electroreduction of CO_2_. *Angew. Chem. Int. Ed.* 2018; **57:** 1944-1948.

3.	Zheng T, Jiang K, Ta N *et al.* Large-scale and highly selective CO_2_ electrocatalytic reduction on nickel single-atom catalyst. *Joule* 2019; **3:** 265-278.

4.	Elouarzaki K, Kannan V, Jose V *et al.* Recent trends, benchmarking, and challenges of electrochemical reduction of CO_2_ by molecular catalysts. *Adv. Energy Mater.* 2019; **9:** 1900090.

5.	Song Y, Chen W, Zhao C *et al.* Metal-free nitrogen-doped mesoporous carbon for electroreduction of CO_2_ to ethanol. *Angew. Chem. Int. Ed.* 2017; **56:** 10840-10844.

6.	Varela A S and Ju WStrasser P. Molecular nitrogen–carbon catalysts, solid metal organic framework catalysts, and solid metal/nitrogen-doped carbon (MNC) catalysts for the electrochemical CO_2_ reduction. *Adv. Energy Mater.* 2018; **8:** 1703614.

7.	Smith P T, Benke B P, Cao Z *et al.* Iron porphyrins embedded into a supramolecular porous organic cage for electrochemical CO_2_ reduction in water. *Angew. Chem. Int. Ed.* 2018; **57:** 9684-9688.

8.	Wang Y-R, Huang Q, He C-T *et al.* Oriented electron transmission in polyoxometalate-metalloporphyrin organic framework for highly selective electroreduction of CO_2_. *Nat. Commun.* 2018; **9:** 4466.

9.	Pan Y, Lin R, Chen Y *et al.* Design of single-atom Co–N_5_ catalytic site: a robust electrocatalyst for CO_2_ reduction with nearly 100% CO selectivity and remarkable stability. *J. Am. Chem. Soc.* 2018; **140:** 4218-4221.

10.	Neese F. The ORCA program system. *Wiley Interdisciplinary Reviews: Comp. Mol. Sci.* 2012; **2:** 73-78.

11.	Schäfer A and Huber CAhlrichs R. Fully optimized contracted Gaussian basis sets of triple zeta valence quality for atoms Li to Kr. *J. Chem. Phys.* 1994; **100:** 5829-5835.

12.	Weigend F, Häser M, Patzelt H *et al.* RI-MP2: optimized auxiliary basis sets and demonstration of efficiency. *Chem. Phys. Lett.* 1998; **294:** 143-152.

13.	Weigend F. Accurate coulomb-fitting basis sets for H to Rn. *Phys. Chem. Chem. Phys* 2006; **8:** 1057-1065.

14.	Grimme S, Antony J, Ehrlich S *et al.* A consistent and accurate ab initio parametrization of density functional dispersion correction (DFT-D) for the 94 elements H-Pu. *J. Chem. Phys.* 2010; **132:** 154104.

15.	Barone V and Cossi M. Quantum calculation of molecular energies and energy gradients in solution by a conductor solvent model. *J. Phys. Chem. A* 1998; **102:** 1995-2001.

16.	Peterson A A, Abild-Pedersen F, Studt F *et al.* How copper catalyzes the electroreduction of carbon dioxide into hydrocarbon fuels. *Energy Environ. Sci.* 2010; **3:** 1311-1315.
